# Heat exposure following encoding can interfere with subsequent recognition memory

**DOI:** 10.1038/s41598-023-38248-w

**Published:** 2023-07-07

**Authors:** Jesús Cudeiro, David Soto, Emilio Gutiérrez

**Affiliations:** 1 Department of Clinical Psychology and Psychobiology, Faculty of Psychology, University of Santiago de Compostela, Santiago de Compostela, Spain; 2 Basque Center On Cognition, Brain and Language, San Sebastian, Spain; 3 Ikerbasque Foundation for Science, Bilbao, Spain; 4 Unidade de Venres Clínicos, Faculty of Psychology, University of Santiago de Compostela, Santiago de Compostela, Spain

**Keywords:** Neuroscience, Psychology

## Abstract

Correlational studies suggest that high temperatures may impair online cognitive performance and learning processes. Here, we tested the hypothesis that heat exposure blocks offline memory consolidation. We report two studies, including a pre-registered replication. First, during a study phase, participants were familiarized with neutral and negatively valenced images. One day later, half of the participants were exposed to high temperatures in a sauna session at 50 °C. Recognition memory was tested 24 h later. We found that participants exposed to high temperature showed an impairment in recognition memory performance, relative to a control group of participants that were not exposed to heat or that had a sauna at 28 °C. This occurred for both emotional and neutral items. These results indicate that heat exposure impairs memory consolidation, thereby opening the possibility of using heat exposure as an agent for the treatment of clinical mental disorders.

## Introduction

In recent years, several observational studies have shown that high temperatures (heat stress) can affect work and school productivity^1–3^. Park et al., showed that heat exposure (> 26.7 °C) during the learning period of students could cause persistent disruptions to the learning process^3^. However, empirical research in this subject is very limited. In animal models, there is some evidence that heat may impair memory consolidation, but this is limited to fish species, worms and flies^4–6^. There are no experimental studies in humans to date that directly evaluate the effect of heat following study on later, offline memory performance.

**Figure 1:**
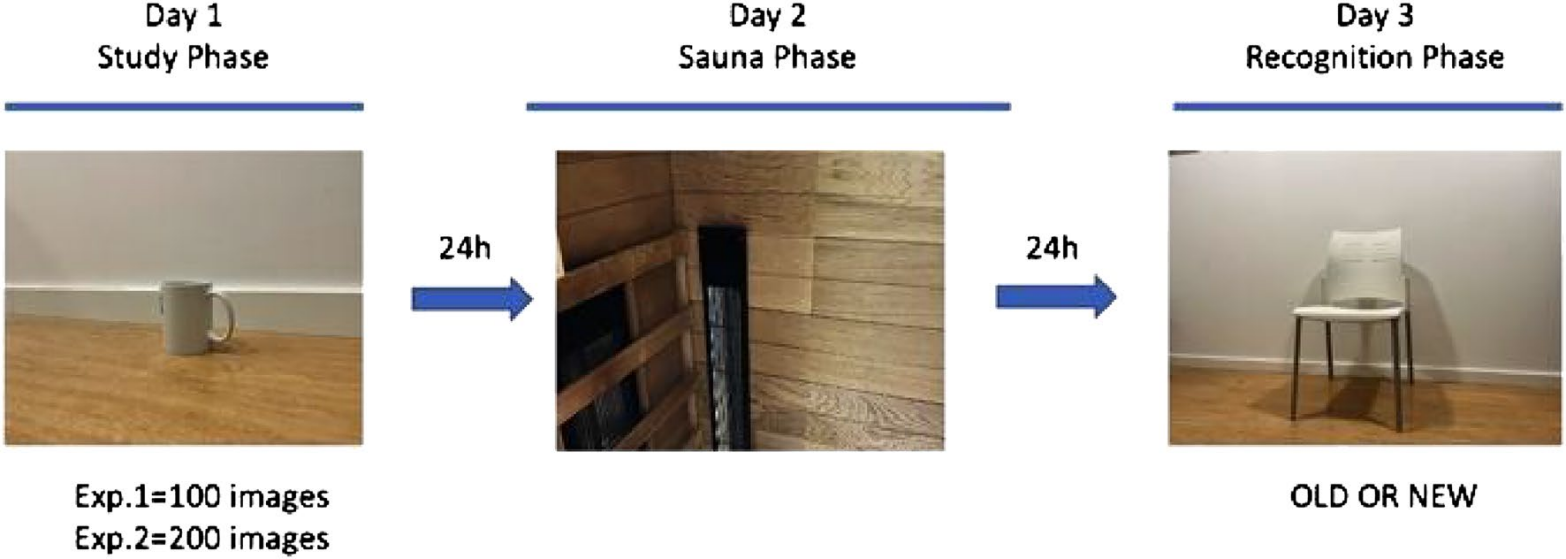
General procedure. In the Study phase, participants learned a number of neutral and emotional images. 24 h later, they had a 7 min sauna session. In Experiment 1 participants underwent the sauna bath at 50 °C or at room temperature (sauna off) while being asked to reactivate images from the previous day or not. One group did not undergo the sauna session. In Experiment 2 all participants had the sauna bath at 50 °C (N = 30) or at 28 °C (N = 30). Twenty-four hours later, all participants completed a recognition memory task.

Our main goal here was to address whether consolidated memories can be disrupted by heat exposure. To do this, we used a variant of the three days paradigm from memory reconsolidation studies^7–9^. Participants first studied a series of emotional and neutral images. Then, 24 h later, the experimental participants are exposed to heat, while the control participants are exposed to a similar environment without heat. To produce heat stress -the condition in which the human body cannot rid of excess heat and the core temperature rise- we used a far-infrared sauna. This type of sauna allows control of temperature rapidly (in a few minutes temperature can rise to 60 °C) and heat penetrates deeply in the body, provoking heat stress in less than 10 minutes^10^. Finally, 24 h later, participants were required to perform a recognition task (see Fig. 1). Initially, we conducted a pilot study assessing the effect of heat exposure and its interaction with memory reactivation on subsequent recognition performance. Evidence from animal models and humans indicates that memory is highly dynamic^11–13^. When a memory is retrieved, it becomes labile for a period of time before being re-stabilized again through a reconsolidation process^14^. Under specific conditions, even consolidated memories can be disrupted. Animal studies showed that reconsolidation can happen when two conditions are present: (1) retrieval of the consolidated memory, (2) blockade of protein synthesis during or shortly after memory retrieval^14,15^. This discovery is used to manipulate maladaptive fear memories in patients with anxiety disorders^16^. In this pilot study, different groups of participants were required (or not) to reactivate (retrieve) the images that had been studied in the previous day while they were exposed (or not) to heat during the sauna bath. We did not observe any effect of reactivation on subsequent recognition performance in the pilot study but there was an effect of heat exposure, impairing on subsequent recognition performance relative to the control group. Then, in a pre-registered replication (pre-registration available at https://osf.io/r3bjg/), we sought to verify the hypothesis that heat exposure can impair the consolidated memory trace of previously studied items.

## Experiment 1: a pilot study

Due to the lack of experimental evidence on the role of heat exposure on the malleability of consolidated memories and subsequent recognition performance^3^, Experiment 1 was an exploratory study the following aims (i) to investigate whether heat exposure affects the memory trace of images that had been previously learned; (ii) assess whether any effect of heat exposure interacts with the emotional valence of the study items and (ii) to test whether the effect of heat interacts with the requirement to reactivate the memory during the sauna. We compared 4 experimental groups: (1) Sauna—Reactivation condition, (2) Sauna—No reactivation condition and (3) No sauna—Reactivation and (4) No sauna—No reactivation.

### Methods

*Participants.* 70 participants (39 females, 31 males; age range = 19–29 years) were recruited from University of Santiago de Compostela via advertisements in the Faculty of Psychology. They were randomly assigned to one of the 4 experimental groups: Sauna—Reactivation (n = 19), Sauna—No reactivation (n = 17), No sauna—Reactivation (n = 18), No sauna—No reactivation (n = 16). All participants provided written informed consent prior to testing. The consent included information about the distressing nature of pictures, recommendations, and risks about the use of sauna (https://sauna.fi/en/) and a reminder that they could end the experiment at any time without any type of penalization. The study conformed to the Declaration of Helsinki. Participants provided informed consent prior to their participation. Ethical approval was obtained from the Comité de Bioética of the University of Santiago de Compostela.

#### Tasks and procedure

*Stimuli.* We used images taken from the International Affective Picture System (IAPS)^17^. We selected 100 pictures—50 neutral and 50 unpleasant—based on standardized scores for emotional arousal and valence (see Supplementary Material). Additionally, 5 more pictures were selected to use as examples for practicing the Study and Recognition phases. To avoid performance biases due to the images, these were subdivided into two sets (i.e., A and B), each consisting of 25 neutral and 25 unpleasant pictures. We performed paired t tests to compare the means of emotional valence between neutral and emotional images of each Set. There were significant differences in set A [M neutral = 5.141, SD = 0.406; M emotional = 2.067, SD = 0.593 [t(24) = 19.943, *p* < 0.001, d = 3.99)] and in set B [M neutral = 4.990, SD = 0.609; M emotional = 2.087, SD = 0.526 [t(24) = 19.331, *p* < 0.001, d = 3.87]. Furthermore, we performed a 2 × 2 ANOVA with Image Set (A, B) × Valence (neutral, emotional) as factors, and found a significant effect of Valence [F(1,48) = 771.43, *p* < 0.001, η_p_^2^ = 0.88] but no effect of Sets [F(1,48) = 0.367, *p* = 0.547] nor interactions between factors [F(1,48) = 0.633, *p* = 0.43]. Half of the participants were presented with set A during study and then presented with set A (old) and B (new) during the recognition task. The other half of the participants were presented with set B in the study phase and the set A were the new items in the test phase. Experimental tasks were programmed with Psychopy^18^.

*State-trait anxiety inventory (STAI)*^19^. The STAI is a 40-item self-report measure designed to evaluate two independent concepts of anxiety: state anxiety (emotional and transitory condition) and trait anxiety (relatively stable anxious propensity). State anxiety was measured in order to test for possible differences across the different groups. The STAI-S consist of a 20-item self-report measure and each item is rated on a 0–3-point scale. Scores range from 0 to 60. We used the Spanish adaptation made by Virella et al.^20^.

Each participant was randomized to one of the four experimental groups (sauna—reactivation group; sauna—no reactivation; no sauna—reactivation and no sauna—no reactivation). All participants performed three experimental sessions (Study Phase, Sauna Phase and Recognition Phase) except for the no sauna—no reactivation group, which only performed the Study and Recognition phases. These sessions were conducted in three consecutive days and always at the same time for each participant. Both the Study and Recognition phase were conducted in the same room while the Sauna Phase took place in a different room equipped with a shower.

*Study Phase* (Day 1). Participants provided written informed consent, as well as the STAI-S. After that, each participant sat at a viewing distance of approximately 70–80 cm. They received the oral and written instruction “In this task you will have to look carefully at a series of images. Before each image there will be a white dot in the center of the screen. Three example images are shown below”. After the presentation of three example pictures, another instruction appeared on the monitor: “when you are ready press space bar”. Each trial lasted 3 s (1 s the fixation dot, 2 s the image). The presentation of the 50 pictures on the Study phase lasted 3 min. During the task, the experimenter remained in the same room with a computer connected to the participant´s monitor. After the Study phase participants rated how they felt “at this very moment” on a visual-analogue scale from 1 (not at all relaxed) to 10 (very relaxed). Before leaving the room, participants were scheduled for the Sauna session 24 h later.

*Sauna phase* (Day 2). *Infrared Sauna (model infraspa TI1)*. One day after the Study phase, the same experimenter received the participants in the sauna room. This had two compartments separated by an opaque barrier with a door, enabling verbal communication between both compartments. The sauna compartment included a dressing space, a shower and an infrared sauna. We used this type of sauna because it allows us to reach a maximum temperature of 60 °C in a few minutes and allows us to program with accuracy the temperature to which the users are exposed. Once there the experimenter explained to each participant the protocol of the sauna session. This protocol included leaving the clothes in the dressing room, taking off eye’s glasses, watches, or jewelry, taking a small towel to sit into the sauna and finally taking a shower after the sauna bath to cool off the body. Participants were advised to avoid alcohol before and after the sauna session. Sauna groups received a 7 min sauna bath at 50 °C which progressively increased to 54/55 °C. This was followed by a shower to lower body temperature. The participants in the Sauna—Reactivation group were explicitly asked to reactivate the memory of the previously studied items by means of the following instruction: “Try to remember as precisely as possible the images you have seen yesterday”. The memory reactivation started at minute 3 of the sauna bath and ended at minute 7. Participants in the sauna without reactivation group followed the same procedure, except that they were not asked to reactivate the memory. The participants in the No sauna—Reactivation group entered the sauna cabin dressed and at room temperature (here the sauna was turned off). They were asked to reactivate memory at minute 3, and they did not take a shower after the sauna bath. The participants in the No sauna—no reactivation group did not undergo the Sauna Phase.

*Recognition phase* (Day 3). Participants performed a recognition memory test in which participants were exposed across trials to the two sets of old and new images (the set they had studied in Day 1 plus the other set of 50 images). In each trial participants had to decide whether the image was old or new and also rate the confidence in the memory decision using a scale ranging from 1 (guess) to 4 (sure). Participants were encouraged to be as accurate as possible without focusing on response time. As in Study Phase, each picture was presented for 2 s after a white fixation point. Subsequently, the old/new decision screen was presented until the response. The experimenter was the same in all four groups for all three days.

### Results

**Figure 2:**
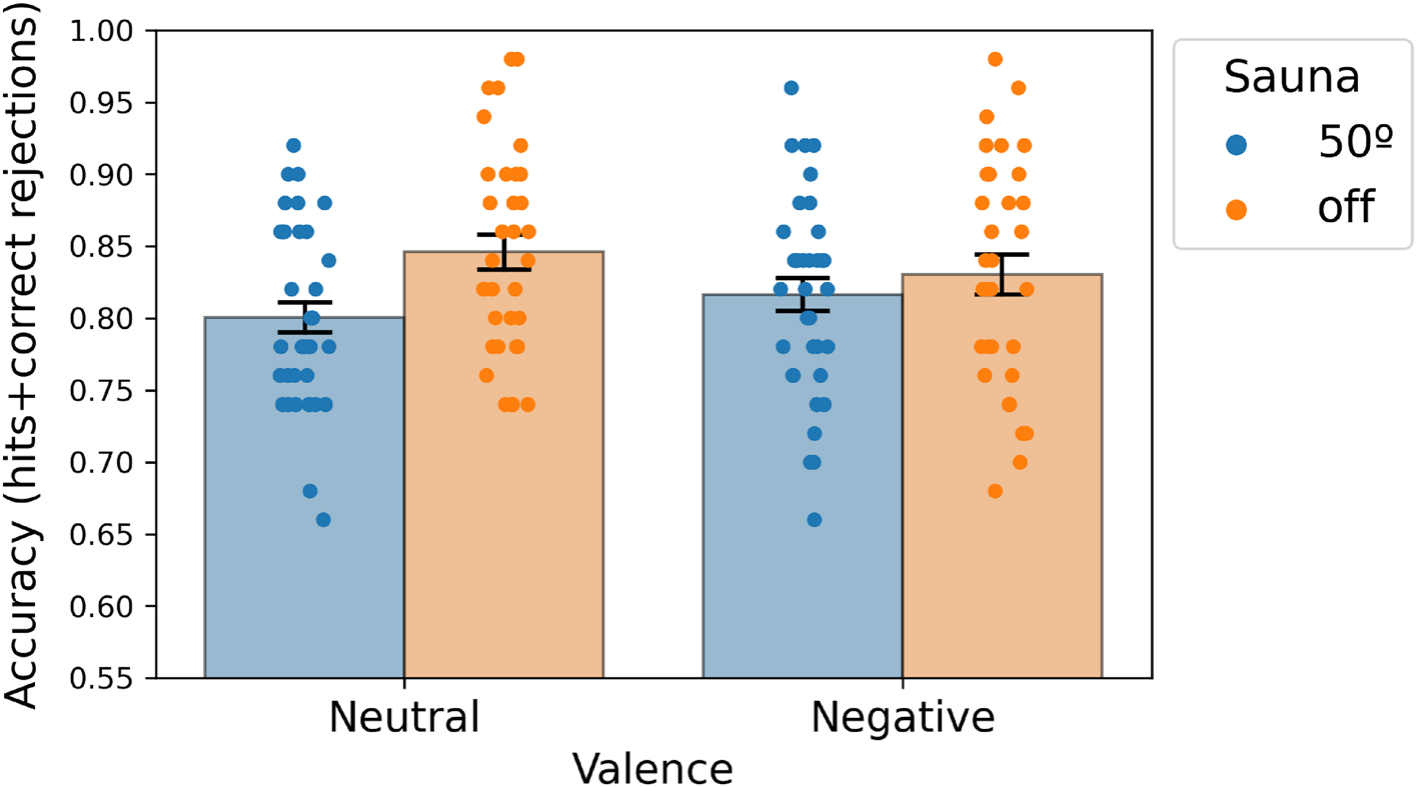
Recognition memory accuracy. This was based on the likelihood of correct responses (i.e. ‘old’ responses to old items and ‘new’ responses to new items) in the recognition memory test. The graph shows data for sauna condition (collapsed across reactivation and no-reactivation groups) and for no sauna condition (collapsed across reactivation and no-reactivation groups).

We performed a mixed ANOVA with two between subject factors (i.e., sauna and memory reactivation) and one within-subject, repeated measures factor (i.e., emotional valence of the images) on the proportion of correct recognition responses in the memory test. Recognition accuracy was operationalised here as the sum of the hit and correct rejection rate in keeping with previous studies^21–23^. Hits were defined as responding ‘old’ when an old item was presented in the recognition test. Correct rejections were defined as “new” responses when a “new” item was presented) The results showed no effect of reactivation on subsequent recognition performance in this pilot experiment [F(1,66) = 2.78; *p* > 0.1], but there was a significant effect of the sauna on day 2 on the subsequent recognition performance on day 3 [F(1,66) = 4.721; *p* = 0.033;* η*_*p*_^2^ = 0.067]. There were no significant interactions between factors, suggesting that the effect was additive across both neutral and negatively valenced images. Figure 2 illustrates the sauna effect on recognition memory as a function of the emotional valence, pooling across the reactivation and the no reactivation condition for the sauna and for the no sauna groups. We did not observe an effect of emotional valence on recognition memory accuracy (*p* = 0.962), however an effect of emotional valence was observed in the hit rate (neutral: M = 0.739, SD = 0.137; negative: M = 0.838, SD = 0.109) [F(1,66) = 39.69; *p* < 0.001; *η*_*p*_^2^ = 0.376], which is in keeping with previous observations in the literature. We also observed an effect of emotional valence on false alarms rates [F(1,66) = 40.547; *p* < 0.001; *η*_*p*_^2^ = 0.183]. There were no further significant interactions between sauna, reactivation and emotion factors on recognition performance (*p* > 0.42). For descriptive statistics see Table 1.

**Table 1:** Descriptive statistics.

	No sauna		Sauna 50 °C	
	Neutral	Negative	Neutral	Negative
A′	.912 ± .044	.892 ± .056	.884 ± .044	.888 ± .051
Hit rate	.799 ± .130	.874 ± .078	.682 ± .121	.803 ± .123
False alarm rate	.107 ± .097	.214 ± .127	.081 ± .072	.171 ± .111

Means and standard deviations of A′, hit and false alarm rates.

To control for potential criterion biases in the recognition test, we re-analysed the data using A′^24^, a bias-free index of memory sensitivity computed based on the hit and false alarm rates (A′ = 0.5 + (0.25 * (((HR − FAR) * (1 + HR − FAR))/(HR * (1 − FAR))). We observed a similar pattern to recognition accuracy, with higher A’ on the no-sauna group (A′ = 0.903) compared to the sauna group (A′ = 0.886), although this effect was marginally significant [F(1,66) = 3.318, *p* = 0.073]. The effect of memory reactivation was not significant [F(1,66) = 2.235, *p* = 0.14, *η*_*p*_^2^ = 0.033].

Further analyses of the mean confidence of recognition decisions did not reveal an effect of sauna (*p* = 0.298), valence (*p* = 0.486) or interactions between factors (lowest *p* value = 0.089). We only observed a main effect of memory reactivation [F(1, 66) = 5.133, *p* = 0.027, *η*_*p*_^2^ = 0.072], with lower confidence in the reactivation group (mean = 2.902) compared to the non-reactivation group (mean = 3.088). Finally, STAI-S scores showed no differences in anxiety state across the different groups [F(1,66) = 1.95; *p* = 0.130].

### Discussion

The results of Experiment 1 showed that recognition of previously studied items was impaired by the administration of a sauna one day after the study phase and one day before the recognition test. To the best of our knowledge, this is the first study assessing the effect of heat in memory re-processing for previously studied information and provides tentative causal evidence for the view that heat exposure impairs learning and performance^3^.

However, the results differ from previous studies as reactivation did not produce any effect on subsequent memory performance. There are several factors that may have contributed to this, including the effects of heat exposure during the sauna or the presence of the experimenter as a reminding cue. One limitation of this study was that the sauna was turned off for those participants in the no sauna-reactivation group who also kept on their clothes at the time of entering the sauna and did not shower afterwards.

Furthermore, literature has shown repeatedly that memory for emotional stimuli and events is more accurate than memory for neutral stimuli and events^25,26^, and we did not observe this memory enhancement for emotional stimuli directly, although we did observe it in the hit rate and false alarm rate. However, this latter effect could be mediated by decision biases and here we were interested in memory sensitivity rather than bias^27^. Hence, we attempted to replicate this finding in Experiment 2 in a more controlled setting in which we manipulated the heat level of the sauna in the experimental and control groups, and we increased the number of images to avoid possible biases in the stimuli selection.

## Experiment 2: A pre-registered replication

Experiment 2 was specifically designed to isolate the effect of heat exposure on recognition memory by directly comparing two groups of participants undergoing sauna at different temperatures. Based on the results of Experiment 1, we predicted that participants undergoing a sauna bath at 50 °C on day 2 will show a reduction in recognition performance on day 3, relative to the control undergoing a sauna bath at 28 °C.

### Methods

*Participants*. 60 participants (44 females, 16 males; age range = 21–29 years) were recruited from University of Santiago de Compostela via advertisements in the Faculty of Psychology. They were randomly assigned to one of the two experimental groups (n = 30): sauna 50 °C, or sauna 28 °C. All participants provided written informed consent prior to testing. The study conformed to the Declaration of Helsinki. Participants provided informed consent prior to their participation. Ethical approval was obtained from the Comité de Bioética of the University of Santiago de Compostela.

*Stimuli and tasks:* The experimental procedure was similar to Experiment 1, with the addition that we selected 100 more images (a total of 200: 100 neutral and 100 emotional images of negative valence). Stimuli were selected from the IAPS, following the basis of their standard scores for emotional arousal and valence. As in Experiment 1, images were subdivided into two sets (A and B), each consisting of 50 neutral and 50 unpleasant pictures. Half of the participants were presented with set A during study and then presented with set A (old) and B (new) during the recognition task. We performed paired t tests to compare the means of emotional valence between neutral and emotional images of each Set. There were significant differences in set A [M neutral = 5.140, SD = 0.777; M emotional = 2.188, SD = 0.529 [t(49) = 22.293, *p* < 0.001, d = 3.153)] and in set B [M neutral = 5.039, SD = 0.71; M emotional = 2.189, SD = 0.519 [t(49) = 22.591, *p* < 0.001, d = 3.195]. Furthermore, we performed a 2 × 2 ANOVA with Image Set (A, B) × Valence (neutral, emotional) as factors, and found a significant effect of Valence [F(1,48) = 1006.492, *p* < 0.001, η_p_^2^ = 0.84]. There were no significant interactions between factors [F(1,48) = 0.25, *p* = 0.616], and there were no significant differences between Sets [F(1,48) = 0.377, *p* = 0.541].

#### Procedure

Experiment 2 aimed to isolate the effect of heat exposure on recognition memory. The reactivation factor from Experiment 1 was not included, and we only compared two groups of participants: sauna 50 °C condition and sauna 28 °C. In this study all participants entered the sauna wearing swimsuits and they took a shower afterwards. The temperature of 28 °C (28.8 °C ± 0.3 °C) was selected because it is considered a value in the thermoneutral range of ambient temperature where the body maintains its core temperature^28^. The experimental procedure across the 3 days was like Experiment 1, including (i) the Study phase on day 1, (ii) the Sauna phase on day 2 and (iii) the subsequent Recognition test on day 3.

### Results

**Figure 3:**
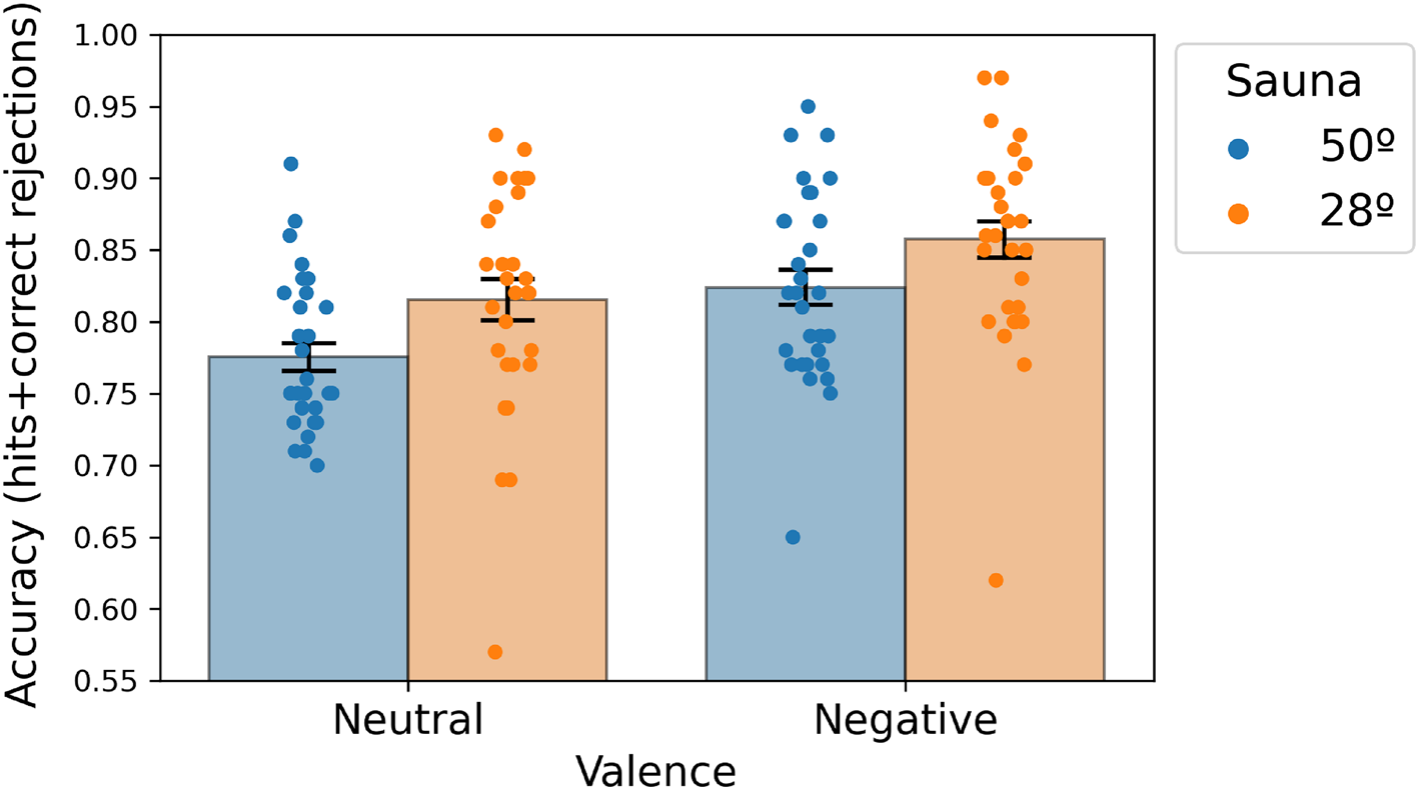
Recognition memory accuracy as a function of the sauna condition and the valence of the items.

We performed an ANOVA with one between subjects factor (Sauna: 28 °C, 50 °C) and one within-subject repeated measures factor (Valence: neutral, emotional) on the proportion of correct responses in the recognition answers. In keeping with Experiment 1, the results depicted in Fig. 3 replicate the significant effect of sauna 50 °C on recognition performance [F(1,58) = 6.438, *p* = 0.014, *η*_*p*_^2^ = 0.1 including all the data; F(1,56) = 14.063, *p* < 0.001, *η*_*p*_^2^ = 0.2, after removing outliers]. The outliers were identified in JASP by using the default values in the Box Plot and label outliers function which basically identifies outliers that that below the Quartile 1 − 1.5 * Inter Quartile Range (IQR) or above the Quartile 3 + 1.5 * IQR. Based on this criterion we removed 3 outliers in Experiment 2. However, for the sake of transparency, we report the results both including and excluding the outliers. In addition, participants showed a better memory for negatively valenced than for neutral stimuli [F(1,58) = 21.108, *p* < 0.001; *η*_*p*_^2^ = 0.267]. There was no significant interaction between sauna and emotional valence (*p* = 0.737). For descriptive statistics see Table 2.

**Table 2:** Descriptive statistics.

	Sauna 28 °C		Sauna 50 °C	
	Neutral	Negative	Neutral	Negative
A′	.885 ± .067	.916 ± .053	.858 ± .043	.893 ± .050
Hit rate	.773 ± .134	.840 ± .107	.742 ± .097	.827 ± .110
False alarm rate	.143 ± .083	.125 ± .093	.191 ± .114	.179 ± .105

Means and standard deviations of A′, hit and false alarm rates.

Finally, to control for potential criterion biases, we conducted the analysis using a bias-free index of memory sensitivity (A′). The effect of heat exposure was also observed in memory sensitivity (50 °C group: A′ = 0.876; 28 °C group: A′ = 0.9) [F(1,58) = 4.732, *p* = 0.034, *η*_*p*_^2^ = 0.075, including all the data; F(1,56) = 14.932, *p* < 0.001, *η*_*p*_^2^ = 0.21, after removing outliers]. STAI-S scores showed no differences in anxiety state between sauna 50 °C group (M = 10.00) and sauna 28 °C group (M = 10.50) [*t*(58) =  − 0.255; *p* = 0.79].

## General Discussion

The goal of this study was to test the effect of heat exposure on memory performance for previously studied information. Experiment 1 showed that the high-temperature sauna manipulation significantly worsened recognition memory one day later. The effect of the heat exposure did not depend on the emotional valence of the items, occurring both for neutral and negatively valence images. A pre-registered Experiment 2 replicated the effect of heat exposure on recognition memory. Notably, the effect of heat exposure on memory performance occurred for items that had been studied a day earlier and hence should be already consolidated in the memory repertoire. These results are in keeping with the view that heat exposure can lead to impairments in learning processes^1–3^. To the best of our knowledge, this is the first causal evidence that inducing an effect of heat exposure by means of a short sauna bath (7 min) can have an impact on the (re-) consolidation of episodic memories in humans.

While the results suggest that the effect of heat exposure on the traces of consolidated memories are independent of the memory reactivation during heat exposure, one may argue that the presence of the experimenter could have acted as a contextual cue and trigger an implicit reactivation even though there was no explicit instruction during the sauna to retrieve the stimuli previously studied on day 1. However, this account is unlikely given that in Experiment 1 there was no evidence that explicit reactivation instructions were associated with better memory performance. Moreover, previous studies have established the need of a reminder trial in order to activate the reconsolidation process, and there is evidence of failures to replicate evidence for the disruption of memory reconsolidation processes^29,30^. Disrupting memory depends on several factors, including the duration of the reminder trial, cue specificity, memory strength and the environmental context in which memory is acquired^31–34^.

Below we provide a tentative neurobiological account of how the effect of heat on memory may come about. Sauna baths involve a considerable thermal load in the body that lead to a massive hormonal reaction. Thermal stress activates hypothalamic–pituitary–adrenal axis, increasing the levels of adrenocorticotropic hormone (ACTH), cortisol, epinephrine and norepinephrine^35^. It is well known that glucocorticoid hormones can modulate consolidation and reconsolidation of memory^36^. However, some evidence indicates that glucocorticoid effects on memory follow an inverted U-shaped curve, as moderate doses improve memory, while higher doses may impair memory^37^. Several studies have shown that administration of cortisone in humans impairs memory retrieval^38,39^. In addition, it has been clearly shown that glucocorticoids reduce hippocampal activation during declarative memory retrieval^40^, and that cortisol suppression (via metyrapone) at encoding^41^ and after memory reactivation weakens emotional memory^42,43^. Thus, it is possible that sauna exposure impairs consolidated memories due to the momentary acute stress produced by hyperthermia, leading to cortisol release and modulation of hippocampal dependent memory networks. However, additional work is needed to test this possibility and make further determinations.

In animal models, the blockade of reconsolidation is achieved by the administration of protein-synthesis inhibitors such as anisomycin^8^, although the use of this type of drugs can be dangerous in humans because of their toxicity. Fortunately, there are alternative behavioral^44–46^ and pharmacological^7,9,47^ agents that are safer to interfere with memory reconsolidation and that opened a window of opportunity to create new approaches of treatment of mental disorders related to anxiety. A recent meta-analysis indicated that behavioral treatments have a small effect in anxiety disorders in comparison to pharmacological treatments^48^. Thus, an important challenge for psychological treatments is to develop more efficacious procedures for interfering memory reconsolidation in a more natural way, without the disadvantages of pharmacological interventions but with sufficient efficacy to allow significant clinical improvements. Heat exposure through a sauna may be a promising tool in this regard but the current paradigm needs to be investigated further in order to develop protocols that can isolate sauna effects on negative, unwanted memories only with ramifications in post-traumatic stress and obsessive–compulsive disorders.

## Supplementary Information

Supplementary Information.

## Data Availability

The behavioral data can be found at https://osf.io/kdh2s/. The pre-registration of Experiment 1 is also available at https://osf.io/r3bjg/.
