# Supplementary material for: Heat exposure following encoding can interfere with subsequent recognition memory
Source: Sci Rep. 2023 Jul 7;13:11024. doi: 10.1038/s41598-023-38248-w (PMC10329023; doi:10.1038/s41598-023-38248-w)
Supplement: Supplementary file 1 — Supplementary Information. [file 41598_2023_38248_MOESM1_ESM.pdf]

Supplementary Material

Image code: pilot study

Examples: 5900, 6370, 9600, 1463, 1300

- Set 1: 1050, 1080, 1121, 1201, 1670, 1908, 2026, 2102, 2190, 2210, 2214, 2383, 2390, 2393, 2513, 2597, 2840, 3000, 3015, 3051, 3059, 3060, 3061, 3063, 3064, 3068, 3100, 3110, 3130, 3131, 3150, 3168, 3220, 3301, 4664.2, 5471, 7002, 7004, 7006, 7040, 7059, 7513, 7546, 8312, 8325, 9253, 9300, 9301, 9320, 9325

- Set 2: 1113, 1274, 1313, 1350, 2104, 2191, 2200, 2230, 2309, 2394, 2400, 2411, 2520, 2590, 2730, 2880, 3010, 3016, 3062, 3069, 3071, 3080, 3102, 3120, 3140, 3170, 3191, 3213, 3261, 3266, 3400, 3530, 3550.1, 6540, 6560, 7009, 7020, 7233, 7235, 7248, 7249, 7250, 7506, 7512, 7550, 7595, 9070, 9302, 9321, 9322, 9468

Image code: replication study

Examples: 5900, 6370, 9600, 1463, 1300

- Set 1: 2025, 2095, 2191, 8130, 7705, 7550, 7505, 7493, 7285, 7236, 7235, 7224, 7195, 7187, 7185, 7183, 7180, 7096, 7055, 7040, 7034, 7025, 7004, 5973, 5661, 5594, 5530, 5500, 5395, 5130, 4536, 4000, 3069, 2980, 2880, 2850, 5973, 2840, 2749, 2745.2, 2514, 2480,2411, 2393, 2345.1, 2230, 2214, 2210, 9270, 9390, 9468, 7053, 9903, 9901, 9635.1, 9490, 9435, 9433, 9332, 9325, 9302, 9300, 9290, 9265, 9254, 9253, 9210, 9183, 9181, 9075, 9040, 9002, 6834, 6821, 6562, 6540, 6520, 6415, 6231, 6212, 6021, 3301, 3225, 3212, 3191, 3181, 3168, 3130, 3110, 3103, 3080, 3064, 3061, 3053, 3030, 3017, 3016, 2981, 2717, 2456

- Set 2: 2026, 2110, 2190, 2206, 2215, 2381, 2383, 2385, 2410, 2575, 2600, 2635, 2702, 2715, 2745.1, 4605, 5455, 5510, 5532, 5740, 5990, 7002, 7010, 7020, 7035, 7050, 7054, 7060, 7077, 7080, 7095, 7160, 7161, 7170, 7175, 7179, 7182, 7186, 7190, 7205, 7217, 7287, 7495, 7503, 7512, 7700, 7710, 8090, 9395, 2811, 3001, 3005.1, 3019, 3051, 3060, 3062, 3063, 3068, 3071, 3100, 3101, 3102, 3120, 3131, 3140, 3150, 3160, 3170, 3185, 3213, 3266, 6022, 6220, 6263, 6350, 6555, 6563, 6825, 6831, 9050, 9070, 9163, 9185, 9187, 9291, 9301, 9320, 9321, 9322, 9326, 9330, 9405, 9410, 9412, 9413, 9414, 9491, 9500, 9900, 2718
